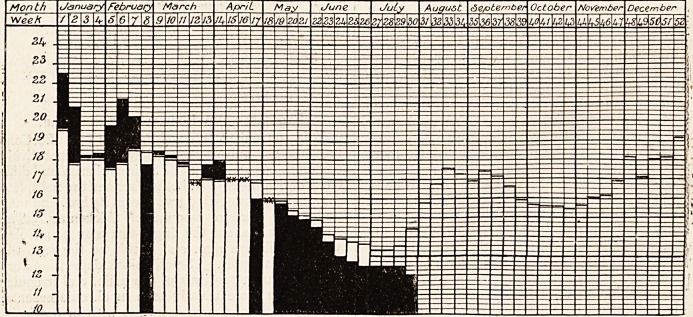# Diagram of the Weekly Death Rate in 1907

**Published:** 1907-08-24

**Authors:** 


### DIAGRAM OF THE WEEKLY DEATH RATE IN 1907.

Showing the weekly death rate for 1907 and the mean weekly death rate for the last Quinquenniad of the 76 great towns of England and Wales.

White columns show mean weekly death rate for last Quinquenniad. Black columns show weekly death rate for current year.

Where death rate for 1907 is in excess of the Quinquennial mean the excess is shown in black above the white column which represents the mean.

Where death rate for 1907 is below the Quinquennial mean the black column is shown in its entire length, the white column which represents the mean, showing above the black.

Where the death rate for 1907 coincides with the Quinquennial mean, it is shown thus xx.

## Figures and Tables

**Figure f1:**